# Clinical and epidemiological aspects of victims of maritime accidents on the Mar Grande-Salvador crossing, Bahia, Brazil: a case series study

**DOI:** 10.1590/1516-3180.2021.0572.R1.14122021

**Published:** 2022-07-15

**Authors:** Danyelle de Souza Mendonça, Paloma Fernandes Carneiro, Adriana Conceição de Mello Andrade, Kátia de Miranda Avena

**Affiliations:** I Undergraduate Student, School of Medicine, Centro Universitário UniFTC, Salvador (BA), Brazil.; II Undergraduate Student, School of Medicine, Centro Universitário UniFTC, Salvador (BA), Brazil.; III MD, MSc. Full Professor, Department of Life Sciences, School of Medicine, Centro Universitário UniFTC, Salvador (BA), Brazil.; IV PT, PhD. Full Professor, Department of Scientific Research, School of Medicine, Centro Universitário UniFTC, Salvador (BA), Brazil.

**Keywords:** Accidental injuries, Asphyxia, Drowning, Epidemiology, Maritime transportation, Maritime accident, Bodily injuries, Intercity waterway transport

## Abstract

**BACKGROUND:**

Maritime transportation is an activity of vital importance for societies. The Mar Grande-Salvador crossing is an intercity waterway transport line in Brazil that transports 3,500 passengers/day, including residents and tourists. In 2017, an accident on this crossing was considered to be the biggest maritime tragedy in Bahia in the last decade.

**OBJECTIVE:**

To describe the clinical and epidemiology characteristics of victims of this maritime accident, with analysis on bodily injuries, causes of death and means/instruments that caused the fatal injuries.

**DESIGN AND SETTING:**

Case-series study at the Forensic Medical Institute of Bahia, Brazil.

**METHODS:**

Reports on 73 victims who were examined for bodily injury or were necropsied by the Forensic Medical Institute were analyzed. This study was approved by the institution’s Research Ethics Committee (protocol 04012218.1.0000.5032).

**RESULTS:**

The victims’ mean age was 33.0 years [95% confidence interval, CI, 26.3-47.0]. The mean age of those who died was 43.0 years [95% CI, 30.5-53.5]. Bodily injuries were found in 74% of the victims. The most frequent bodily injuries were ecchymoses among females (69.7%) and abrasions among males (76.2%). Blunt instruments produced most bodily injuries (85.2%). Among the victims who died, 68.4% were female. Mechanical asphyxiation through drowning was the leading cause of death (89.4%). The overall lethality rate was 26%, and this was higher among females (28.2%).

**CONCLUSION:**

Women were the main victims of this maritime accident. Bodily injuries occurred more frequently than death, but these injuries proved to be quite significant, thus demonstrating the importance of measures to improve the safety of navigation.

## INTRODUCTION

Maritime transportation is an activity of vital importance to societies.^1^ However, this activity is associated with several risks, among them the possibility of accidents, which can cause marine pollution and loss of life.^2^

According to the Brazilian Navy, between January and August 2017, 107 shipwrecks were recorded in the country, a number 12.6% higher than the number of cases that occurred in the same period of 2016.^3^

Among the maritime accidents recorded in 2017, the one that occurred on the Mar Grande-Salvador crossing can be considered to have been the biggest maritime tragedy in Bahia in the last decade and the second biggest in Brazil that year, second only to the accident in the Xingu River, in the state of Pará, which left 21 dead. The Mar Grande-Salvador crossing is an intercity waterway transport line in Brazil that crosses the bay known as “Baía de Todos os Santos” to connect the island of Itaparica to the mainland. On average, 3,500 passengers are transported per day, including both residents and tourists.^4^

Over the years, measures have been taken to improve safety in shipping, such as regulations and crew training. However, navigation accidents remain a source of concern^5^ due to the number of events and fatalities.^6^

In shipwrecks, one of the concerns is the drowning of passengers and crew members. About 500,000 people per year die worldwide as a result of such events.^7^ Drowning can happen in several circumstances, and the cases most often reported occur in fresh water. However, in coastal areas, accidents involving navigation and water sports are common.^8^

In Brazil, deaths due to drowning exceed 5,700 cases/year, and most of the victims are children. In 2016, about 89% of drownings in Brazil had unintentional causes. Of these, 1.1% were due to boating accidents.^9^

Death due to drowning is a three-phase process: defense, resistance and exhaustion. The defense phase can be further divided into surprise and dyspnea. In the resistance phase, as a form of defense, breathing movements are interrupted. This is followed by the exhaustion phase, when resistance ceases and the victim starts breathing deeply and then loses consciousness and dies.^10^ Individuals who survive the submersion episode may develop late complications resulting from water aspiration, such as severe infections and pulmonary edema, which may later result in the victim’s death.^11^

In accidents in fluvial or marine waters, when an unconscious person is found in shallow waters, it needs to be considered that spinal cord injury, head trauma or sudden illness may have occurred. These can be caused by acute myocardial infarction, convulsion, lipothymia, hydrocution (thermal shock) or primary drowning in which the victim ends up in shallow waters.^12^ Given that head trauma is responsible for 25% of trauma deaths, this should be evaluated among individuals who are victims of accidents involving water.^13^

Maritime accidents can result not only in drowning, but also in other injuries to the human body. According to the Brazilian Penal Code, these may be classified as mild, serious or very serious, and may also involve intentional injuries.^14^ From the medical-legal point of view, intentional injuries are consequences of a violent act that is capable of directly or indirectly producing damage to someone’s physical integrity or health, or that is responsible for worsening or continuation of an already existing disturbance. These injuries therefore consist of alterations to the biopsychosocial balance. Violence should be understood not simply as the consequence of mechanical action, but as the means of action, whether physical, chemical, physicochemical, biochemical, biodynamic or mixed.^15^

Given the large number of shipwrecks recorded over recent years and the need for research describing maritime accidents that occurred in Brazilian territory, it is relevant evaluate the possible causes of death and bodily injury among the victims. Drowning may or may not have been the ultimate cause. Thus, there is a need to assess whether drowning is, in fact, what kills the most during this type of accident.^16^

## OBJECTIVE

In this context, considering the proportions of this accident on the Mar Grande-Salvador crossing, the aim of the present study was to describe the clinical and epidemiological characteristics of the victims and to analyze their bodily injuries, the cause of death and the means or instrument of action that produced the fatal injuries.

## METHODS

This was a case series study conducted through analysis on reports from the Forensic Medical Institute of Bahia, Brazil. These were obtained through the Integrated System for Forensics Administration of the Technical Police Department of Bahia.

The study population was composed of the 116 passengers and four crew members of the express ferry “Cavalo Marinho I”, which suffered a maritime accident during the Mar Grande-Salvador crossing on August 24, 2017. This express ferry departed from the Mar-Grande Maritime Terminal, on the island of Vera Cruz, heading for Salvador. Its capacity was 160 passengers.

The study population included people of both sexes and all ages (children, young people, adults and elderly people) who had suffered bodily injuries or died as a result of this maritime accident. There were no exclusion criteria in this study since the objective was to characterize the epidemiology of all the victims of the shipwreck.

The variables of the study were age, sex and the existence of bodily injury or death. Among the victims who suffered bodily injury, we also evaluated: (I) types of bodily injury; (II) whether the injury resulted in harm to the bodily integrity or health of the victim; (III) what the instrument or means that produced the injury was; (IV) whether the injury caused incapacity for normal activities for more than thirty days; and (V) whether the injury resulted in danger to life, permanent debilitation of a limb or impairment of its sensation or function, acceleration of labor, permanent incapacity to work, incurable disease, loss or disablement of a limb or loss of its sensation or function, permanent deformity or abortion. Among the victims who died, we evaluated: (I) the cause of death; (II) the instrument or means that produced the fatal injury or injuries; and (III) whether poison, fire, explosives, asphyxiation, torture or other insidious or cruel means were used, or the means resulted in common danger, or whether any means that made it difficult or impossible to defend the victim was used.

Data analysis was performed using the IBM SPSS software, version 26.0 (SPSS Inc., Chicago, Illinois, United States). Categorical variables were presented as the frequency distribution of the categories, represented as absolute numbers (n) and percentages (%). Numerical variables were described as medians and 95% confidence levels. In addition, the lethality rate was calculated overall and according to sex.

Because this was a study involving human beings, it was submitted to and approved by our institution’s research ethics committee on Dec 12, 2018, in accordance with the ethical principles of the Helsinki Declaration and with Resolution 466/2012 of the Brazilian National Health Council. This study was registered through authorization certificate number 04012218.1.0000.5032.

## RESULTS

An overview of the accident revealed that there was a total of 73 victims, who were all among the passengers on the vessel. The distribution of the victims with bodily injuries and deaths according to age and sex is shown in Table 1.

**Table 1.** Distribution according to age and sex among the victims with bodily injuries and the deaths due to the maritime accident on the Mar Grande-Salvador crossing, Bahia, Brazil, in 2017

Victims	n (%)	Age (years)		
		MD [95% CI]	Minimum	Maximum
**Bodily injuries**				
Female	33 (61.1)	34.0 [25.0-47.0]	19	75
Male	21 (38.9)	31.0 [27.0-47.0]	21	65
**Total**	**54 (100.0)**	**33.0 [26.3-47.0]**	**19**	**75**
**Deaths**				
Female	13 (68.4)	48.0 [38.0-53.0]	20	70
Male	6 (31.6)	18.5 [2.0-59.0]	0.5	68
**Total**	**19 (100.0)**	**43.0 [30.5-53.5]**	**0.5**	**70**

n = frequency; MD = median; CI = confidence interval.

Among the victims, 54 people (74%) had bodily injuries, and these were predominantly females (61.1%). Their median age was 33.0 years [95% confidence interval, CI, 26.3-47.0]. By stratifying them according to sex, it was observed that the women who suffered bodily injuries were older than the men (34.0 years [95% CI, 25.0-47.0] and 31.0 years [95% CI, 27.0-47.0], respectively).

There was a mortality rate of 26%, totaling 19 fatal victims, who were predominantly female (68.4%); their median age was 43.0 years [95% CI, 30.5-53.5]. The lethality rate among females was higher than among males (28.2% versus 22.2%, respectively). In addition, the women who died were older than the men (48.0 years [95% CI, 38.0-53.0] versus 18.5 years [95% CI, 2.0-59.0], respectively).

Regarding the cause of death, it was observed that mechanical asphyxia due to drowning occurred in 89.4% of the deaths (n = 17). The remaining deaths occurred due to brain hemorrhage and mechanical asphyxia after head trauma (n = 1; 5.3%) and to an undetermined cause (n = 1; 5.3%).

From evaluating the instrument or means that produced the injury, it was found that a physical-chemical means was the main agent responsible for the deaths, totaling 84.2% of the cases (n = 16). A physical-chemical means in association with blunt instruments resulted in 10.5% of the deaths (n = 2). In 5.3% of the cases (n = 1), the analysis was impaired.

Considering the instruments or means that produced bodily injuries, the most prevalent type was blunt instruments, which accounted for 85.2% of the injuries produced (n = 46). Injuries caused by blunt/short blunt instruments and blunt instruments/physical-chemical means accounted for 3.7% (n = 2) each.

Regarding the bodily injuries presented by the victims, it was observed that among women, ecchymoses were most prevalent, in 69.7% of the cases, followed by abrasions in 66.7% and contusion wounds in 24.2%. Unlike the results found among females, abrasions were the most prevalent injuries among males, totaling 76.2% of the cases, followed by ecchymoses in 38.1% and contusion in 19.0%. The other injuries presented are described in Table 2. It is important to note that combinations of different types of bodily injuries were present in some of in the accident victims.

**Table 2.** Distribution of bodily injuries according to sex among the victims of the maritime accident on the Mar Grande-Salvador crossing, Bahia, Brazil, in 2017

Bodily injuries^*^	Victims**	
	Female (n = 33)	Male (n = 21)
Ecchymosis	23 (69.7)	8 (38.1)
Excoriations	22 (66.7)	16 (76.2)
Contused wound	8 (24.2)	4 (19.0)
Edema	3 (9.1)	1 (4.8)
Cutting wound	2 (6.1)	0 (0.0)
Cutaneous contusion lesion	1 (3.0)	1 (4.8)
Hemorrhagic effusion in right eye	0 (0.0)	1 (4.8)
Absence of lesion	1 (3.0)	0 (0.0)

* Presence of bodily injury in associated or unassociated form; **frequency (percentage).

In assessing the consequences of the injuries suffered by the victims, it was found that in 1.9% of the cases there was incapacity in relation to the usual occupations for more than 30 days, while 85.2% did not suffer any such incapacity. For 92.6% of the victims, their bodily injuries did not result in any life-threatening condition. In addition, 85.2% of the victims did not suffer any permanent weakness of limb or impairment of its sensation or function, or any acceleration of labor, while 13.0% relied on further examinations to find out whether the weakness would be permanent or not (Table 3).

**Table 3.** Distribution of the consequences of injuries suffered by victims of the maritime accident on the Mar Grande-Salvador crossing, Bahia, Brazil, in 2017

Consequences*	Victims (n = 54)			
	IPO	Danger to life	PWL	PDW
Yes	1 (1.9)	2 (3.7)	0 (0.0)	0 (0.0)
No	46 (85.2)	50 (92.6)	46 (85.2)	46 (85.2)
Depending on examinations	6 (11.1)	2 (3.7)	7 (13.0)	7 (13.0)
Damaged	1 (1.8)	0 (0.0)	1 (1.8)	1 (1.8)

IPO = inability to perform usual occupations for more than thirty days; PWL = permanent weakness of limb or impairment of its sensation or function, or acceleration of labor; PDW = permanent disability to work, incurable disease, loss or disablement of limb or loss of its sensation or function, permanent deformity or abortion; *frequency (percentage).

## DISCUSSION

In this study, we present the clinical-epidemiological profile, characteristics of bodily injuries, cause of death and the means or instrument of action that produced the injuries of the 73 victims of the maritime accident that occurred during the Mar Grande-Salvador crossing, in Bahia, Brazil, on August 24, 2017.

In this maritime accident, females were more prevalent among the victims who died. This finding differed from what was reported in a study conducted by Araújo et al.,^17^ who showed that most of the fatal victims of drowning were male. According to Pinheiro Júnior et al.,^18^ men are more adventurous and expose themselves to greater risk than do women; and moreover, women usually have less physical conditioning. These factors favor the results presented by Araújo et al.^17^ It is noteworthy that while the study by Araújo et al.^17^ analyzed all the cases of drowning in the city of Ribeirão Preto, state of São Paulo, between the years 2001 and 2004, the present study analyzed the cause of death and the means or instrument of action that produced the fatal injuries, through necropsies performed on the victims of a marine accident. Thus, the circumstances of the deaths were different.

Regarding the age of the fatal victims, only three children died in this accident: two were two years old and one was six months old. Corroborating this finding, the study by Quan and Cummings,^19^ carried out between 1980 and 1995, showed that children between 0 and 4 years old, when alone, tend to drown when they fall into pools or open waters (rivers or lakes), while people between 35 and 64 years old are usually sailing when drowning occurs.

In the maritime accident that occurred on the Mar Grande-Salvador crossing, the main cause of death was mechanical asphyxiation through drowning. According to Armstrong and Erskine,^20^ drowning is considered, in most cases, to be an asphyxiating process that can cause systemic repercussions in several organs, and the lungs are certainly the organ most affected during this process.^7^ As a consequence of the obstruction caused either by foreign bodies or by fluid aspiration, hypoxia (decreased oxygen in the organ/tissue), hypoxemia (decreased oxygen in the blood) and asphyxia appear. Subsequently, if the obstruction is not reversed, neuronal injury and cardiorespiratory arrest ensue.

Head trauma can occur through any injury resulting from a blunt or penetrating force to the head that leads to involvement of vessels and meninges, and loss of consciousness is a possible consequence of this process.^12,21^ In addition, mental confusion, convulsion and focal deficit may be symptoms associated with head trauma.^13^ In our study, we found that only one passenger (5.3%) died due to brain hemorrhage and mechanical asphyxia after head trauma. Drowning can be attributed as a secondary mechanism resulting from encephalic lesion, which, through compromising the level of consciousness, makes the victim unable to remain on the water surface, thus causing subsequent drowning.

Regarding bodily injury, injuries produced by blunt instruments generate blunt injuries and these include abrasions, ecchymoses and hematomas.^22^ Factors such as age, sex, location of the injury and fragility of blood vessels can influence the victim’s susceptibility to contusion. These factors may explain the higher prevalence of blunt instruments in producing the bodily injuries suffered by the accident victims, as well as the higher prevalence of bruises and excoriations.

The women who were victims of the maritime accident on the Mar Grande-Salvador crossing presented more blunt injuries than did the men. According to Vanezis,^23^ women commonly present greater susceptibility to developing bruises due to their greater deposition of subcutaneous fat, which favors the appearance of such findings, thus corroborating the findings of the present study.

According to the Brazilian Penal Code (item I, Paragraph 1, Article 129), serious bodily injury can produce consequences such as incapacity to do one’s habitual occupations for more than 30 days, permanent weakness of the limb or impairment of its sensation or function, and acceleration of labor. On the other hand, the consequences of very serious injuries include permanent incapacity to work, incurable disease, and loss or disablement of the limb or loss of its sensation or function.

In analyzing this accident, we noticed that most of the injuries were not considered severe or very severe. This corroborates the findings of Verdan,^24^ who stated that bruises and scratches are considered to be minor injuries.

The high lethality rate demonstrated in the present study highlights the severity of this maritime accident. However, despite the large number of shipwrecks that happen in Brazil, the number of studies addressing this subject is very small. Thus, there is a need to encourage further research in this field.

## CONCLUSION

This study showed that injuries such as ecchymosis and abrasions were the ones most commonly found among the victims, and the main instruments that produced these injuries were blunt instruments. Moreover, it was observed that the main cause of death was mechanical asphyxiation through drowning, and that physical-chemical media were mostly responsible for causing lethal trauma.

Women were the main victims of this maritime accident, in relation to both bodily injuries and deaths. However, it was not possible to determine how many men and how many women in total were present on the boat, and this can be considered to be a limitation of this study, since women may have been more affected because they were present in larger numbers on the boat.

Accidents involving boats and ships occur widely around the world, and are responsible for large numbers of deaths through drowning. The present study is relevant in that it brings specific data from a maritime accident and thus contributes support for strategies aimed towards prevention of injuries in such accidents.

It is essential to develop measures for improving the safety of navigation, such as training for the crew, safety policies that encourage the use of life jackets and, lastly, stimulation of vessel inspections, so that the number of deaths can be minimized.

## References

[1] Teixeira AP, Antão P, Soares CG, Soares CG, Teixeira AP, Antão P (2005). Análise e Gestão de Riscos, Segurança e Viabilidade.

[2] Santos MGF (2013). Análise de acidentes com embarcações em águas sob jurisdição brasileira: uma abordagem preventiva.

[3] Gomes D (2017). Perigo nas águas. CNT Transporte Atual..

[4] Gonçalves AP, Queiroz PS (2019). Abordagem sistêmica para investigar trágico acidente marítimo no Brasil. Laborare..

[5] Chauvina C, Lardjane S, Morel G, Clostermannc JP, Langarda B (2013). Human and organisational factors in maritime accidents: Analysis of collisions at sea using the HFACS. Accid Anal Pre..

[6] Haurelhuk SS (2017). Segurança da navegação: percepção das capitanias fluviais, dos portos e do Tribunal Marítimo.

[7] Chandy D, Weinhouse GL (2019). Drowning (submersion injuries).

[8] Salomez F, Vicent JL (2014). Drowning: a review of epidemiology, pathophysiology, treatment and prevention. Resuscitation..

[9] Szpilman D, Webber J, Quan L (2014). Creating a Drowning Chain of Survival. Resuscitation..

[10] Szpilman D (2019). Sociedade Brasileira de Salvamento Aquático.. Manual de Emergências aquáticas.

[11] Porcides AJ, Coordenadoria Estadual de Defesa Civil do Paraná (2006). Manual do Atendimento Pré-Hospitalar do Corpo de Bombeiros do Paraná.

[12] Papadodima SA, Athanaselis SA, Skliros E, Spiliopoulou CA (2010). Forensic investigation of submersion deaths. Int J Clin Pract..

[13] Oliveira E, Lavrador JP, Santos MM, Antunes JL (2012). Traumatismo cranioencefálico: Abordagem Integrada. Acta Med Port..

[14] Sabino M (2017). Lesão corporal: Particularidades e características. Boletim Informativo Criminológico..

[15] França GV (2015). Medicina Legal.

[16] Peden MM, McGee K (2003). The epidemiology of drowning worldwide. Int J Inj Control Saf Promot..

[17] Araújo RT, Martin CCS, Martinis BS, Evison MP, Guimarães MA (2008). Dados médico-legais sobre afogamentos na região de Ribeirão Preto (SP- Brasil): Um passo para a prevenção. Medicina (Ribeirão Preto).

[18] Pinheiro ML, Tabosa EMC, Viana MCC (2012). Perfil clínico e epidemiológico de pacientes vítimas de afogamento no município de Fortaleza/CE. Rev Saude Publ (Florianópolis)..

[19] Quan L, Cummings P (2003). Characteristics of drowning by different age groups. Inj Prev..

[20] Armstrong EJ, Erskine KL (2018). Investigation of Drowning Deaths: A Practical Review. AFP Journal..

[21] Brasil. Ministério da Saúde (2015). Diretrizes de atenção à reabilitação da pessoa com traumatismo cranioencefálico.

[22] Grego R, Douglas W (2016). Medicina Legal: À Luz do Direito Penal e do Direito Processual Penal.

[23] Vanezis P (2001). Interpreting bruises at necropsy. J Clin Pathol..

[24] Verdan TL (2012). Analisando o Crime de Lesões Corporais: Uma Breve Apreciação. Rev Cien Sem Acad (Fortaleza).

